# Supplementary material for: Causal Relationship between Aging and Anorexia Nervosa: A White-Matter-Microstructure-Mediated Mendelian Randomization Analysis
Source: Biomedicines. 2024 Aug 16;12(8):1874. doi: 10.3390/biomedicines12081874 (PMC11351342; doi:10.3390/biomedicines12081874)
Supplement: Supplementary file 1 [file biomedicines-12-01874-s001.zip › Supplementary Figure/Figure S8/Leaveoneout plot of MR analysis for TL to ubm-b-1492.pdf]

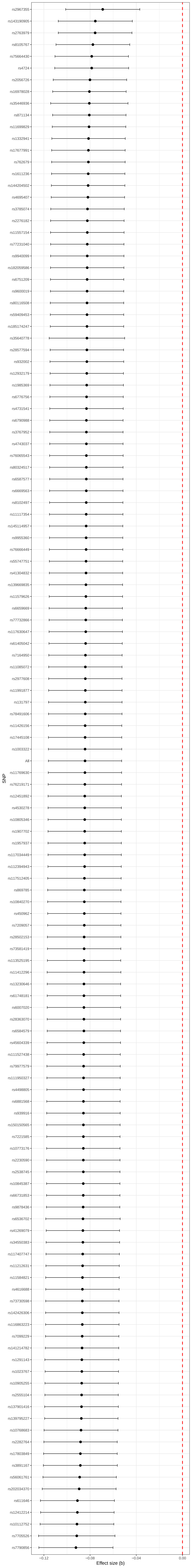
rs2967355
rs143190905
rs2763979
rs8105767
rs75664430
rs4724
rs2056726
rs16978028
rs35446936
rs871134
rs11699829
rs1332941
rs17677991
rs762679
rs1611236
rs144204502
rs4695407
rs3785074
rs2276182
rs11557154
rs77231040
rs9940099
rs182059586
rs6751209
rs9600019
rs80116508
rs59409453
rs185174247
rs35640778
rs28577594
rs932002
rs12932179
rs1985369
rs6776756
rs4731541
rs6790988
rs3767952
rs4743037
rs76065543
rs80324517
rs6587577
rs6669563
rs8102497
rs11117354
rs145114957
rs9955360
rs76666449
rs55747751
rs41304832
rs139669835
rs11579626
rs6659669
rs77732866
rs117630647
rs61405042
rs7164950
rs11085072
rs2977608
rs11991877
rs131797
rs78491606
rs11426156
rs17445108
rs1003322
All
rs11769630
rs76219171
rs12451892
rs4530278
rs10805346
rs1907702
rs1957937
rs117034449
rs112394943
rs117512405
rs869785
rs10840270
rs450962
rs7209057
rs28502153
rs73581419
rs113525195
rs11412296
rs13230646
rs61748181
rs6007020
rs28363070
rs6584579
rs45604339
rs111527438
rs79977579
rs111950327
rs4498805
rs6881568
rs939916
rs150150565
rs7221585
rs10773176
rs2230590
rs2538745
rs10845387
rs66731853
rs9878436
rs6536702
rs41269079
rs34550363
rs117407747
rs12212631
rs11584821
rs4616688
rs73730598
rs142426306
rs116863223
rs7099229
rs141214782
rs1291143
rs1023767
rs10905255
rs2555104
rs137901416
rs139795227
rs10768683
rs2282764
rs17803849
rs3891167
rs56061761
rs202034370
rs611646
rs12412214
rs10112752
rs7705526
rs7790856
SNP
-0.12
-0.08
-0.04
0.00
Effect size (b)
